# Sucrose diffusion in aqueous solution

**DOI:** 10.1039/c6cp03238a

**Published:** 2016-06-21

**Authors:** Hannah C. Price, Johan Mattsson, Benjamin J. Murray

**Affiliations:** a School of Earth and Environment , University of Leeds , Leeds , UK . Email: hannah.price@ncas.ac.uk; b School of Physics and Astronomy , University of Leeds , Leeds , UK . Email: k.j.l.mattsson@leeds.ac.uk

## Abstract

The diffusion of sugar in aqueous solution is important both in nature and in technological applications, yet measurements of diffusion coefficients at low water content are scarce. We report directly measured sucrose diffusion coefficients in aqueous solution. Our technique utilises a Raman isotope tracer method to monitor the diffusion of non-deuterated and deuterated sucrose across a boundary between the two aqueous solutions. At a water activity of 0.4 (equivalent to 90 wt% sucrose) at room temperature, the diffusion coefficient of sucrose was determined to be approximately four orders of magnitude smaller than that of water in the same material. Using literature viscosity data, we show that, although inappropriate for the prediction of water diffusion, the Stokes–Einstein equation works well for predicting sucrose diffusion under the conditions studied. As well as providing information of importance to the fundamental understanding of diffusion in binary solutions, these data have technological, pharmaceutical and medical implications, for example in cryopreservation. Moreover, in the atmosphere, slow organic diffusion may have important implications for aerosol growth, chemistry and evaporation, where processes may be limited by the inability of a molecule to diffuse between the bulk and the surface of a particle.

## Abstract 2

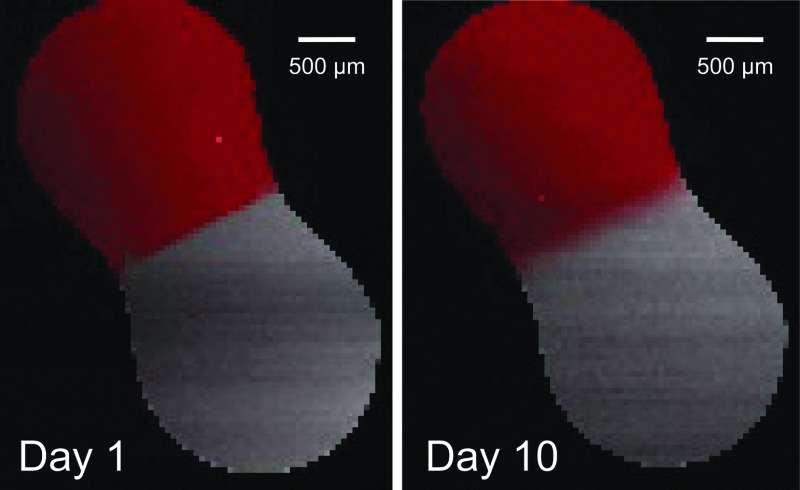
We report the first direct measurements of sucrose diffusion in aqueous solution at sucrose mass fractions above 0.75.

## 1 Introduction

Aqueous solutions of sugars such as sucrose are abundant in nature. They have important roles in the metabolism of organisms as energy sources and structural agents, and can protect from freezing or dehydration in extreme environmental conditions.^1–3^ Sugar solutions also have important technological applications in food preservation and in the cryopreservation of proteins or cells^4–6^ and they are commonly used in pharmaceutical formulations where they provide a matrix for storage and controlled release of active components.^5,7^ In the atmosphere, aerosol particles composed of aqueous solutions respond to changes in the surrounding relative humidity (RH) by taking up and losing water in a process known as hygroscopic growth. This process governs atmospheric visibility and cloud formation, and its study is thus of vital importance to the understanding of our climate.

Of key importance to the roles played by aqueous sugar solutions is the diffusion of molecules within sugar-based low moisture materials. In the cryopreservation of biological matter or in the preservation of foods, for instance, the diffusion of oxygen or metabolants can strongly affect the viability of a particular formulation.^5,8^ In the atmosphere, slow diffusion within aqueous organic aerosol particles has been suggested to affect heterogeneous chemistry, whereby molecules at the centre of a particle are effectively shielded from gas phase oxidants.^9,10^

In some aqueous sugar solutions, solute crystallization at high solute concentration or low temperature can be inhibited due to an increase in viscosity and associated decrease in the rate of diffusion within the solution as it supersaturates or supercools.^11^ The dynamics in a glass-forming liquid generally involve several molecular relaxations, typically termed α, β and γ *etc.* in order of increasing characteristic relaxation frequency. The α, or primary, relaxation is the mechanism behind structural relaxation and is directly related to glass formation. The other higher frequency secondary relaxations typically persist within the glassy state. When the molecular motion, as characterized by the structural α-relaxation time *τ*_α_, slows down so that *τ*_α_ surpasses the longest experimental equilibrium time-scale, typically ∼100 s, an out-of-equilibrium solid – a glass – is formed. This glass has the physical properties of a solid but lacks the long-range molecular order of a crystal. The glass-transition temperature or concentration is commonly defined where *τ*_α_ = 100 s. An increase in *τ*_α_ corresponds to an increase in viscosity *η* and using the Maxwell relation *τ*_α_ = *η*/*G*_∞_,^12,13^ the glass transition corresponds to a viscosity of ∼10^12^ Pa s, since the instantaneous shear modulus *G*_∞_ is typically ∼1–100 GPa and only weakly temperature dependent.^13^ Thus, the glass transition is often alternatively defined where *η* = 10^12^ Pa s.

Near the glass transition, *η* and *τ*_α_ are highly dependent on temperature. Some materials show close to Arrhenius behaviour, *i.e. η* = *η*_0_ exp(*E*_A_/*k*_B_ *T*), where *η*_0_ and *E*_A_ are constants and denote the high temperature limit of the viscosity and the activation energy, respectively. These materials are termed “strong” and are characterised by an activation energy which is either independent or very weakly dependent on temperature. Materials which show a clear non-Arrhenius temperature dependence of *η* or *τ*_α_ are termed “fragile” and could be viewed as having an effective activation energy that increases with decreasing temperature.^14^ In a dynamic range corresponding to temperatures above *T*_g_, *T* ∼ (1.2–1.6)·*T*_g_, several changes in the liquid dynamics are generally observed. These changes include a cross-over to a different temperature dependence for the structural α-relaxation and the merging of the α-relaxation with a secondary so-called β-relaxation which is active in the glassy state. There is significant evidence suggesting that a secondary relaxation is a generic feature of glass-formation, and that the α- and β-relaxations are generally coupled.^15–18^ Aqueous sucrose solutions show both α- and β-relaxations.^19^

In a glass-forming liquid far above its dynamic cross-over range, the shear viscosity *η* is typically inversely related to the translational diffusion coefficient, *D*, according to the Stokes–Einstein (SE) relation:1
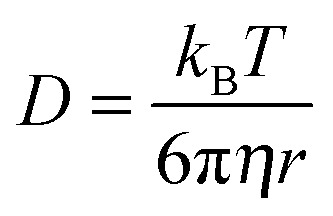
where *k*_B_ is the Boltzmann constant, *T* is the temperature, *η* is the viscosity and *r* is the radius of the diffusing entity. The SE-relation describes the self-diffusion of a spherical probe particle in a viscous continuum fluid and the derivation is based on classical hydrodynamics combined with kinetic theory.^20,21^ In the highly fluid state above the dynamic cross-over, the SE-relation often holds well even if the probe particle becomes very small, and even self-diffusion of the fluid molecules themselves generally follow the SE-relation.^22,23^ However, as a fluid approaches its glass transition, the SE-relation generally under-estimates *D* relative to *η* by as much as several orders of magnitude; this is often referred to as a breakdown of the SE-relation. Instead of the SE behaviour *D* ∝ *η* ^–1^, a fractional dependence on *η* develops where *D* ∝ *η* ^–*ζ*^, and *ζ* < 1. The observed values of *ζ* are normally situated in the range ∼0.6–0.9^23–26^ and have been reported to vary with the fragility of the liquid.^27,28^ Moreover, in the dynamic range where the fractional SE is observed, a significant probe size dependence of the translational diffusion is generally found, where the deviation from SE behaviour is more pronounced for smaller probes.^29–31^

The breakdown of the SE relation and the emergence of a fractional SE have been observed not only for molecular liquids,^29–33^ but for a wide range of fluids in computer simulations^23,24,34–36^ and for colloidal systems.^35,37^ Very similar behaviour has also been observed for diffusion controlled crystal growth where the growth rate ∝*η* ^–*ζ*^ and *ζ* was shown to vary with the fragility of the liquid.^28,38^ Both the SE breakdown and the fractional SE are thus commonly observed, which suggests that they correspond to generic or at least very general behaviour of glass-forming systems.

Given this generality, it is important to ask how multi-component systems behave as their glass transition is approached. For aqueous sugar solutions it has been observed that SE does not seem to hold at high sugar concentrations and 3–6 orders of magnitude separation between the time-scales characterising diffusion and those predicted by the SE relation have been reported.^25,39–43^ There are, however, very few data sets available over wide concentration or water activity ranges and our understanding of the breakdown of SE in these types of systems is thus relatively poor. Very similar results to those reported for aqueous mixtures have been found also for multi-component metallic glassy alloys^44–47^ for which the diffusion behaviour of individual atomic species of different size were found to vary significantly. As an example, a recent study on a metallic glass-forming alloy found that the larger atomic species followed the SE relation even in the deeply supercooled range, whereas the smaller atomic species decoupled dramatically and showed 4 orders of magnitude difference from the SE prediction based on their size.^44,46,47^ For binary systems consisting of spherical particles with significant size disparity both theoretical studies based on mode coupling theory^48–51^ and computer simulations^51,52^ have demonstrated the possibility of a significant decoupling between the dynamics of the smaller and larger particles. Similar effects have also been observed for binary colloidal suspensions^51,53^ and for binary mixtures of glass-forming liquids and oligomers or polymers.^54,55^

The most common explanation for the SE breakdown is that it corresponds to the onset of dynamical heterogeneities (DH) as the glass transition is approached. DH consist of spatial regions in the fluid characterised by significantly different relaxation times and the development of DH in the deeply supercooled state is a general feature of glass-forming systems.^22,27,56^ One often-suggested link between DH and the SE breakdown is that the latter occurs due to differences in how self diffusion and viscosity are averaged over the underlying distribution of characteristic time-scales.^22,29^ This interpretation has recently been questioned based both on experimental and computer simulation observations.^46,47,57^ Other suggested explanations instead propose that the SE breakdown arises due to differences in how translational diffusivity and viscosity (or structural relaxation) couple to spatially varying intermolecular cooperativity, and/or due to a link between translational diffusion and the secondary β-relaxation mechanisms that emerge as the glass transition is approached.^45,47^ It is clear that we do not fully understand diffusion near and within the glassy state, and this is particularly emphasised for multi-component glass-forming systems.

In this work, we address the paucity of information regarding the diffusion of organics in highly concentrated aqueous sugar solutions by directly measuring sucrose diffusion in aqueous sucrose. Our study allows for an assessment of the applicability of the SE description and provides detailed information about the SE breakdown for sucrose solutions at low water activites. The results of this study are thus of direct relevance for atmospheric aerosol, food preservation and cryoprotection of biological matter, for which the formation of highly viscous aqueous solutions are key.

## 2 Experimental

Large organic molecules are expected to diffuse more slowly in aqueous solutions than small molecules such as water. To measure the diffusion coefficient of sucrose molecules in aqueous solution, a Raman isotope tracer method was employed which was similar to that described by Zhu *et al.* ^40^ The diffusion of sucrose molecules across a boundary between aqueous solutions of deuterated and non-deuterated sucrose was monitored by virtue of the differing wavenumber locations of the C–D and C–H Raman stretch bands. All raw Raman spectra, humidity and temperature data used in this work are provided as a dataset in Price *et al.* ^92^

### 2.1 Experimental setup

Aqueous solutions of 33 wt% sucrose (Sigma, >99.5%) and deuterated sucrose (β-d-[UL-^2^H_7_]fructofuranosyl α-d-[UL-^2^H_7_]glucopyranoside, Omicron Biochemicals) were made using Milli-Q (18.2 MΩ cm) pure water. A droplet of each solution was placed on a hydrophobic siliconised glass slide (Hampton Research) using a micropipette, and put in a temperature and humidity controlled cell in a Renishaw inVia Raman microscope system equipped with a 514 nm laser. The droplets were then allowed to equilibrate with the surrounding water vapour. Because molecular diffusion in aqueous sucrose depends on water content, and the purpose of this experiment was to measure diffusion coefficients at pre-determined water concentrations, it was important that a uniform water activity across each droplet was achieved. In order to achieve a water activity of RH/100, the time taken for equilibration was calculated using previously measured water diffusion coefficients for aqueous sucrose,^58^ together with a multi-shell water diffusion model.^59^ At RHs below 50%, this step was performed at an elevated temperature (up to 36 °C) to speed up equilibration time (as discussed by Price *et al.* ^58^).

**Fig. 1:**
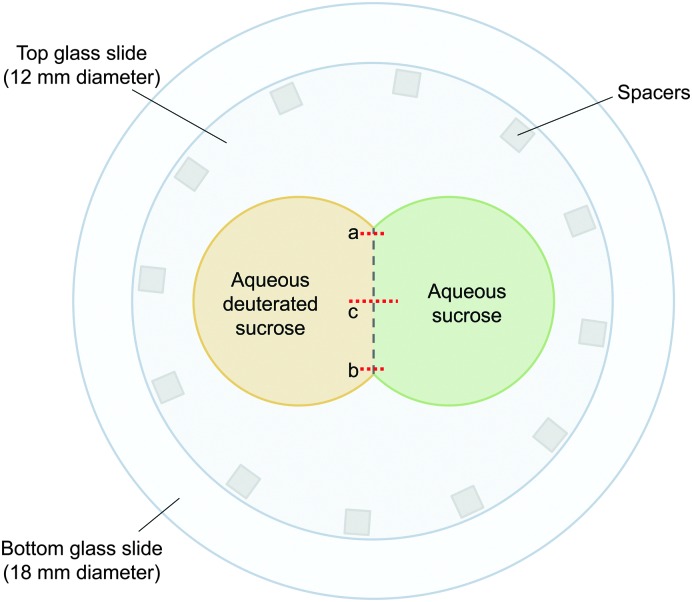
Setup used for measuring sucrose diffusion in aqueous sucrose solutions. The top glass slide causes the two droplets (one non-deuterated sucrose, the other deuterated sucrose) to make contact, and is held in place by spacers.

Once a uniform water activity across each droplet radius had been achieved, the RH controlled cell was briefly opened, with the humidified N_2_ still flowing over the sample, to allow a second hydrophobic siliconised glass slide to be place on top of the droplets. This slide was prepared by placing several squares of double-sided adhesive tape around its edge to act as spacers and prevent slippage. By applying a small amount of force to this top slide, the two droplets were compressed and made contact, as illustrated in Fig. 1.

**Fig. 2:**
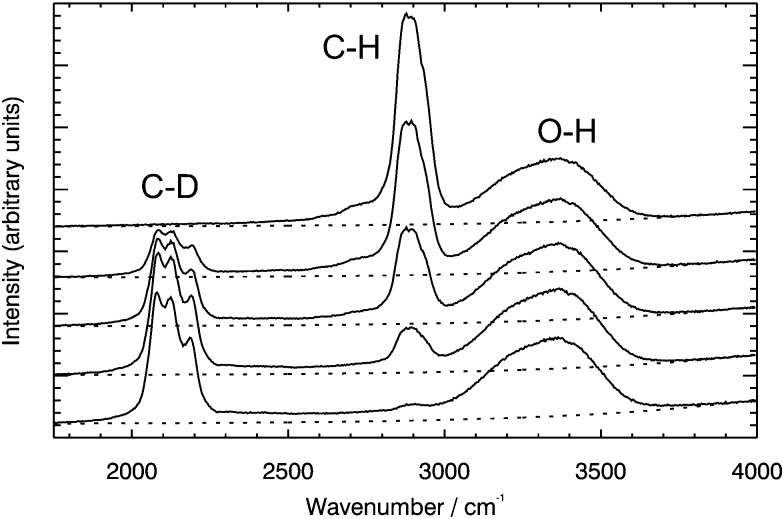
Raw Raman data for aqueous sucrose showing the gradual decrease in the C–H stretch (at 3800 to 3100 cm^–1^) band and increase in the C–D stretch (at 2000 to 2300 cm^–1^) band as the Raman laser traces a path across the boundary between the deuterated and non-deuterated sucrose droplets. The unchanging band at 3100 to 3600 cm^–1^ is the O–H stretch. The dotted lines show the Gaussian curve used for the background subtraction.

Raman measurements were made to monitor the progress of sucrose (both non-deuterated and deuterated) diffusion across the boundary. The high-wavenumber Raman spectrum of non-deuterated aqueous sucrose features an O–H stretch band at ∼3100 to 3500 cm^–1^, and a C–H stretch band at ∼2800 to 3100 cm^–1^. The spectrum of deuterated aqueous sucrose lacks the C–H stretch band, and instead has a C–D band at ∼2000 to 2300 cm^–1^. Five Raman spectra taken along a track traversing the boundary between the deuterated and non-deuterated solutions are shown in Fig. 2, with the decrease in C–H and increase in C–D bands clearly visible.

### 2.2 Diffusion across a plane interface

Assuming the boundary between the deuterated and non-deuterated solutions can be treated as a semi-infinite plane at the spatial co-ordinate *x* = 0, the intensities of the C–H band, *I*_*h*_, is described by:^40,60^
2
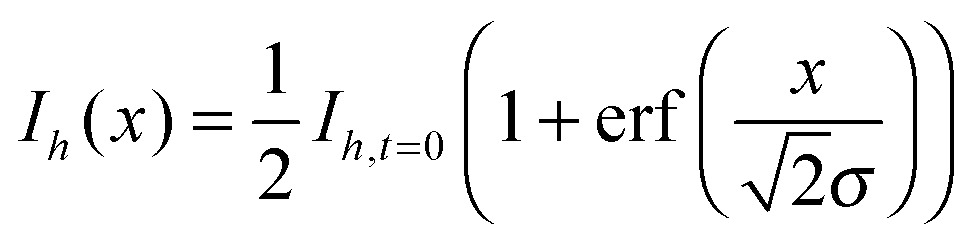
where *I*_*h*,*t*=0_ is the intensity of the C–H stretch in aqueous non-deuterated sucrose and *σ* describes the width of the interface broadened by diffusion. Similarly,3
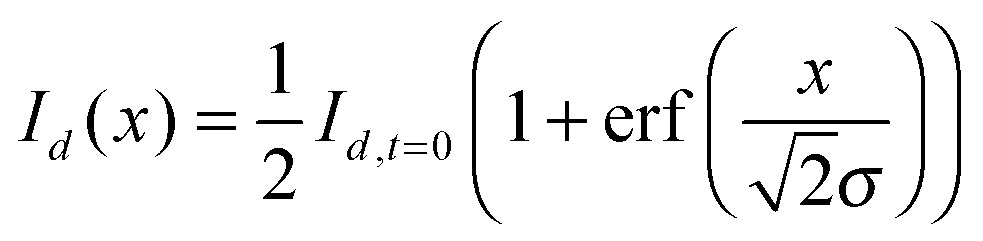
where *I*_*d*,*t*=0_ is the intensity of the C–D stretch in aqueous deuterated sucrose.

To use eqn (2) and (3) to determine the diffusion coefficient of sucrose, it was necessary to make Raman measurements along a perpendicular bisector to the boundary between the different solutions. To determine the location of this line, two short series of spectra were taken at either end of the boundary, marked as (a) and (b) in Fig. 1. The points at which the C–H and C–D intensities were the same in each of these series were used to find the location of the boundary, and trigonometry was used to determine the position of the perpendicular bisector, marked as (c) in Fig. 1. A series of spectra were acquired along this line with a spatial separation and acquisition time chosen such that the duration of the collection of this series was short in comparison to the diffusion timescale.

**Fig. 3:**
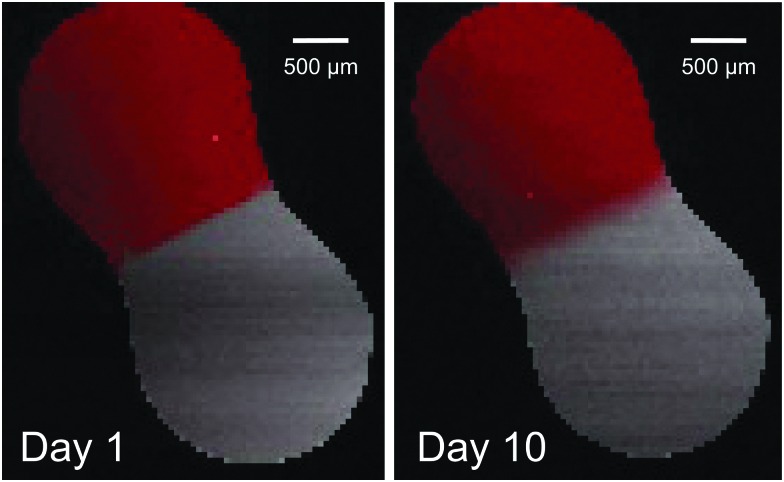
Raman maps of an aqueous non-deuterated and aqueous deuterated sucrose droplet, in contact at 60% RH. The red colour corresponds to the background-corrected intensity of the C–H stretch band, and the grey colour corresponds to the background-corrected intensity of the C–D stretch band. On intial contact (day 1), the boundary between the deuterated and non-deuterated regions is sharp. By day 10, this boundary has been broadened by diffusion.

Fig. 3 shows a map of the Raman band intensities of the C–H (red) and C–D (grey) stretches. At the start of the experiment, the non-deuterated and deuterated aqueous solutions were in contact, but diffusional mixing of sucrose molecules had not yet occurred: the change from C–H to C–D between the two droplets was abrupt. As time progressed, diffusional mixing gradually caused a blurring of the boundary between the two droplets, seen by a more continuous change in colour from red to grey.

### 2.3 Analysis of Raman spectra to determine organic diffusion coefficients

At the start of each experiment, a series of spectra were acquired along the perpendicular bisector to the boundary between the deuterated and non-deuterated sucrose solutions, as described above. The background was subtracted from each spectrum by fitting a Gaussian curve plus a constant to the regions where no C–H, C–D or O–H peaks were present, using the Levenberg–Marquardt technique.^61^ This Gaussian curve was constrained in wavenumber and width using a fit to a spectrum taken of the background (the slide without the samples). Each spectrum was normalised to the background-corrected intensity of the (constant) O–H band.

**Fig. 4:**
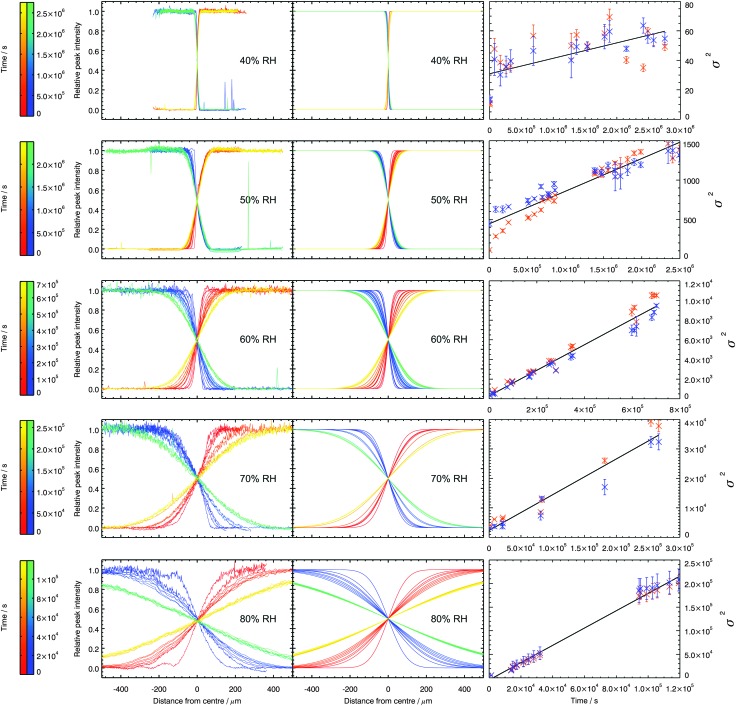
Background corrected, normalised peak intensities of the C–H (red to yellow) and C–D bands (blue to green), relative to their maxima, as time progresses after initial contact between the deuterated and non-deuterated droplets. Experimental data is shown on the left, and the fits to this data in the middle. The plots on the right show the temporal evolution of the interfacial width, with the fitted lines used to calculate the diffusion coefficient. Orange datapoints correspond to *σ* ^2^ values calculated based on the evolution of the C–H peak; blue datapoints correspond to *σ* ^2^ values calculated based on the evolution of the C–D peak.

After a time interval (defined by the rate of diffusion – at high RHs this was around half an hour; at low RHs this was a day) the series of spectra was collected again, and this was repeated as the interface broadened. The broadening of the boundaries between the non-deueterated and deuterated regions as time progressed is shown on the left hand side of Fig. 4. Each curve was fitted according to eqn (2) and (3), in order to determine the width of the interface, *σ*. These fits are shown in the middle column of Fig. 4.

The diffusion coefficient of sucrose, *D*_sucrose_, was determined *via* the temporal evolution of *σ* with time:

4 *σ*^2^ = *σ*_*x*_^2^ + 2*Dt*

where *σ*_*x*_ is the interfacial width due to the instrument's spatial resolution and *t* is the time since contact was made between the two droplets. The gradient of a line fitted to *σ* ^2^ *vs. t* is therefore double *D*_sucrose_. These lines are shown on the right in Fig. 4. The error in each *D*_sucrose_ measurement was calculated using the linear regression standard error in that gradient.

## 3 Results and discussion

### 3.1 Sucrose diffusion coefficients

**Fig. 5:**
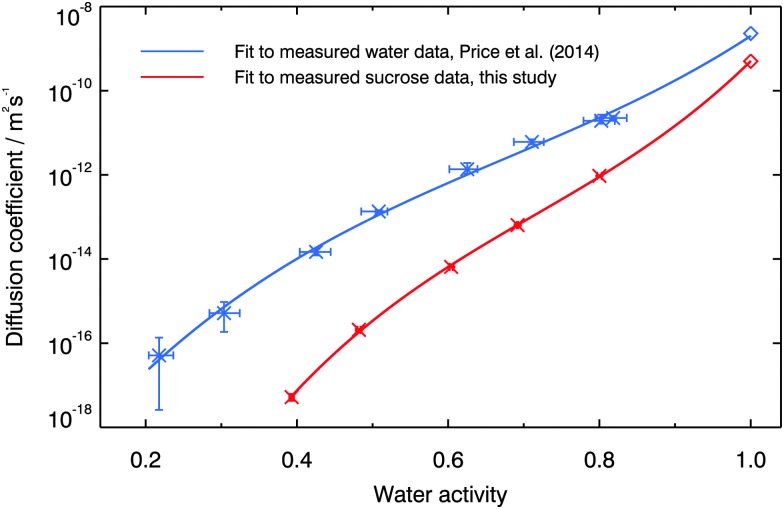
Measured diffusion coefficients of sucrose and water^58^ in aqueous sucrose at 296 K, as a function of water activity. The diffusion coefficients of water and sucrose in water at a water activity of 1.0 are shown using diamonds.^62,63^ Solid lines are empirical fits to the data, according to eqn (5). Note that error bars are shown for both sets of data but are considerably smaller for the sucrose diffusion coefficients because of the different experimental setup. Uncertainties in water activity are larger for the water diffusion experiments because they incorporate the difference in vapour pressure between normal and heavy water. Since only H_2_O was used for the sucrose diffusion experiments, the error bars are much smaller.

The measured diffusion coefficients of sucrose in aqueous solution are shown *vs.* water activity in Fig. 5. Also shown for comparison are the measured water diffusion coefficients in the same material.^58^ Lines are fitted to the data as follows:

5 log_10_ *D* = *a* + *ba*_w_ + *ca*_w_^2^ + *da*_w_^3^

where *a*, *b*, *c* and *d* are empirically fitted parameters detailed in Table 1. The fits for all substances converge to the diffusion coefficient for water and sucrose in water as water activity tends to 1.0.^62,63^ It can be seen that the diffusion of sucrose is slower than that of water at any given water activity, with the difference between the two increasing as water activity decreases.

**Table 1:** Fit parameters *a* to *d* used in eqn (5) for *D*_sucrose_(*a*_w_) in aqueous sucrose, valid at water activities above 0.4. Also shown are the fit parameters for *D*_water_(*a*_w_) in the same material, reproduced from Price *et al.*,^58^ valid at water activities above 0.2

	*a*	*b*	*c*	*d*
Water	–20.89	25.92	–26.97	13.25
Sucrose	–30.97	54.89	–62.34	29.12

**Fig. 6:**
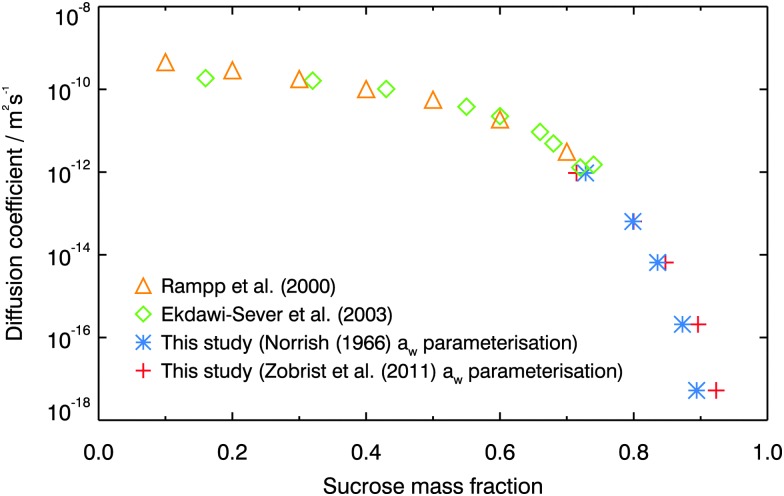
Diffusion coefficients of sucrose measured using Raman isotope tracer method at 296 K, compared with NMR measurements of sucrose diffusion by Rampp *et al.* ^64^ and Ekdawi-Sever *et al.* ^65^ To compare diffusion coefficients on the same scale, water activity was converted to sucrose mass fraction using either the Norrish^66^ or the Zobrist *et al.* ^43^ parameterisation.

Fig. 6 shows how the sucrose diffusion coefficients measured in this study compare with those measured at lower concentration using NMR.^64,65^ The literature data were reported in terms of sucrose mass fraction, whereas the experimental setup used here was designed to quantify sucrose diffusion at a given water activity. Water activity is therefore converted to sucrose mass fraction for the purposes of this plot, using the two different parameterisations given by Norrish^66^ and Zobrist *et al.* ^43^ Regardless of which parameterisation is used, the high sucrose mass fraction diffusion coefficients measured in this study follow on smoothly from the lower sucrose mass fraction literature diffusion coefficient data. In the small region where the three datasets overlap, all measured sucrose diffusion coefficients are in good agreement.

**Fig. 7:**
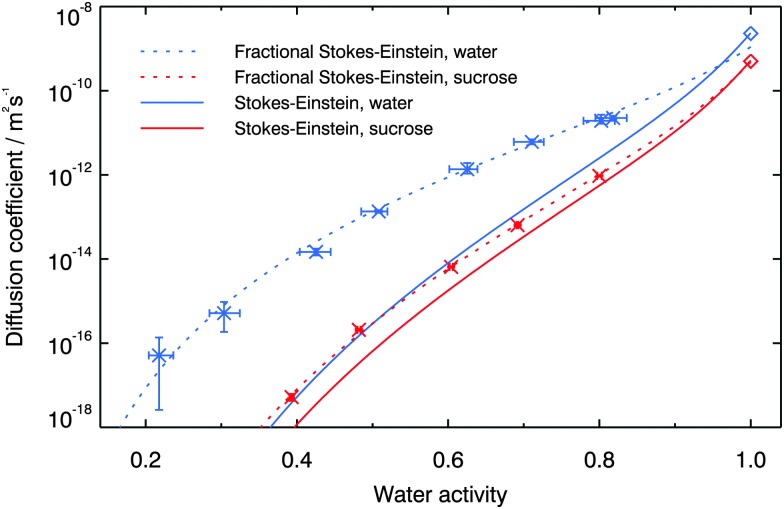
Measured diffusion coefficients compared with Stokes–Einstein predictions. The diffusion coefficients of water and sucrose in water at a water activity of 1.0 are shown using diamonds.^62,63^ The solid lines show the predicted diffusion coefficients of water and sucrose calculated based on the viscosity parameterisation given by Chenlo *et al.*,^68^ using the thermodynamic treatment for water activity of Norrish.^66^ The dotted lines show two fractional Stokes–Einstein relationships, *i.e. D* = *Cη* ^–*ζ*^ where *C* = 2 × 10^–11^ and 9 × 10^–13^ and *ζ* = 0.57 and 0.90 for water and sucrose, respectively.

As discussed earlier, the Stokes–Einstein equation that relates diffusion to viscosity is known to break down under certain conditions. Power *et al.* ^67^ measured the viscosity of highly concentrated aqueous sucrose solutions at room temperature over a range of different RHs. They found that a line closely fitting their data could be produced by using the viscosity parameterisation given by Chenlo *et al.*,^68^ when RH (or water activity) is converted to molal concentration using the thermodynamic treatment for water activity of Norrish.^66^ By linking water activity to viscosity in this way, we compute diffusion coefficients of water and sucrose in aqueous sucrose using the Stokes–Einstein relation, shown in Fig. 7. The molecular diameters used in the Stokes–Einstein equation were 2 Å for water and 9 Å for sucrose (calculated based on the density of amorphous sucrose given by Hancock and Zografi^69^). At a water activity of 0.6 the relation underpredicts water diffusion by a factor of ∼100, and at a water activity of 0.4 this underprediction increases to a factor of ∼3000. Much better agreement, however, is found for sucrose diffusion. At a water activity of 0.4 (where the uncertainty in the viscosity measurements of Power *et al.* ^67^ are a factor of ∼4) the diffusion coefficient of sucrose is underpredicted by the relation by a factor of ∼6. The Stokes–Einstein equation is thus much better able to predict sucrose diffusion than water diffusion, at least over the range of water activities studied here. This is in broad agreement with earlier work in a range of materials using molecular probes of differing sizes^29–31,44–47^ showing that larger molecules diffuse more slowly than small molecules.

**Fig. 8:**
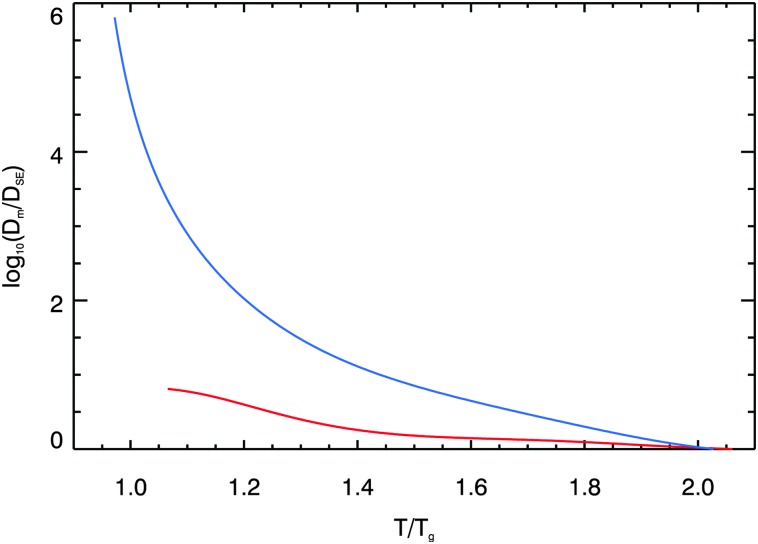
The (logarithm of the) ratio of the diffusion coefficient parameterisation based on the Raman isotope tracer methods to that predicted using the Stokes–Einstein (*ζ* = 1) preditions for water and sucrose, plotted as a function of *T*/*T*_g_. Viscosities used in these predictions were calculated according to the parameterisation given by Chenlo *et al.*,^68^ using the thermodynamic treatment for water activity of Norrish.^66^

The breakdown of the Stokes–Einstein relationship is further demonstrated in Fig. 8, where the discrepancy between the diffusion coefficient parameterisations based on direct measurements are compared with the Stokes–Einstein predictions as a function of *T*/*T*_g_. *T*_g_ was calculated for aqueous sucrose solutions across the water activity range studied using the parameterisation given in Zobrist *et al.* ^70^ At 296 K, the temperature at which our experiments were performed, the glass transition occurs at a water activity of approximately 0.25. Deviations from Stokes–Einstein behaviour have typically been observed previously for *T*/*T*_g_ ∼ 1.5, but here it can be seen that by this point the water diffusion coefficient in aqueous sucrose is already being underpredicted by an order of magnitude. It is clear from the figure that the Stokes–Einstein relationship breaks down near to the glass transition for both sucrose and water diffusion, but is vastly more pronounced in the case of water diffusion.

Although the Stokes–Einstein relationship is shown here not to hold at low water activity, it is intriguing to note that a fractional Stokes–Einstein relationship can describe both the water diffusion and sucrose diffusion, as is demonstrated in Fig. 7. For sucrose solutions, as for other solutions, the fragility might be expected to vary with water concentration and thus water activity.^11,71,72^ As discussed in the introduction, *ζ* is believed to vary with fragility. It is thus interesing that over the relatively wide range of water actitivies studied here we can describe the behaviour using a fixed value of *ζ*. Water diffusion can be described using a fractional behaviour with a lower *ζ* and sucrose diffusion with a higher, but in both cases we can use a fixed *ζ* over the full water activity range.

**Fig. 9:**
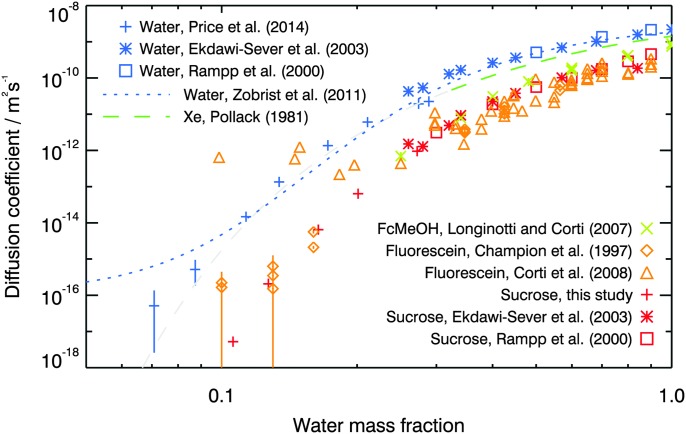
Diffusion coefficients of water,^43,58,64,65^ sucrose,^64,65^ fluorescein,^39,41^ ferrocene methanol^75^ and xenon^25^ in aqueous sucrose. Colour corresponds approximately to the size of the diffusant: the largest, sucrose, is shown in red, followed by fluorescein in orange, then ferrocene methanol in yellow-green, xenon in blue-green and finally water, the smallest, shown in blue. Water activities used in this study and reported by Zobrist *et al.* ^43^ and Price *et al.* ^58^ are converted to water mass fraction using the parameterisation given by Norrish.^66^ The grey dashed line represents an extrapolation of the Pollack^25^ parameterisation. We include this more speculative regime on the basis that the parameterisation is arrived at *via* a straight line fit in log(diffusion coefficient) *vs.* log(viscosity) space; the agreement with the Price *et al.* ^58^ water diffusion coefficient data is interesting.

Literature values of the diffusion coefficients of water and carbohydrate molecules in aqueous solution have been determined previously at lower solute concentrations using NMR,^64,65^ shown in Fig. 9. Rampp *et al.* ^64^ observed that the ratio of the diffusion coefficient of water to the diffusion coefficient of sucrose, α,α-trehalose, allosucrose and leucrose in aqueous solutions increased as temperatures decreased, and speculated that this was because the water molecules were able to diffuse through a hydrogen-bonded network formed by the carbohydrate molecules. Computational studies have suggested that water and carbohydrate molecules diffuse differently in concentrated aqueous solutions, where simulations indicate that the diffusion of carbohydrates is continuous whilst water molecules are able to make random jumps.^65,73,74^ The Stokes–Einstein description is based on macroscropic hydrodynamics and assumes the material to be a contiuum. The differences in diffusion mechanism between water and carbohydrates could therefore provide an explanation for the differing degrees to which it underpredicts the water and sucrose diffusion coefficients presented here.

Also shown in Fig. 9 are diffusion coefficients of fluorescein (measured using fluoresence recovery after photobleaching (FRAP) techniques by Champion *et al.* ^39^ and Corti *et al.* ^41^) and of ferrocene methanol (measured using an electrochemical method by Longinotti and Corti^75^) in aqueous sucrose. The discrepancy between the two sets of fluorescein results at high concentrations could be due to sample preparation (for example Corti *et al.* ^41^ added sodium hydroxide to their sucrose solutions to increase the pH to ∼8), or the restricted experimental duration which limited the region of the FRAP recovery curve which could be fitted by Champion *et al.* ^39^ Except for the measurements of Corti *et al.* ^41^ below a water mass fraction of 0.2, the three datasets are similar to our sucrose diffusion coefficients and there are no large deviations from Stokes–Einstein behaviour observed within error under these conditions. This good agreement with Stokes–Einstein behaviour could be due to the similarities in molecular diameters between the diffusants and the major component of the solution, sucrose: fluorescein has a molecular diameter of ∼7 Å,^76^ whilst ferrocene methanol has a molecular diameter of ∼4.5 Å.^77^

Interestingly, the measured diffusion coefficients of water^43,58,64,65^ and xenon^25^ are similar. Water and xenon are close in size (water has a molecular diameter of ∼2 Å and xenon has an atomic radius of 1.08 Å), and so may be expected to diffuse at similar rates, but again the degree to which they diffuse faster than sucrose can not be explained solely by the Stokes–Einstein equation. Effects such as interactions between the diffusant and the host solution, the degree to which H-bonding is important, and differences in diffusion mechanism all have effects beyond the simplicity of the hydrodynamic description of the Stokes–Einstein equation.

### 3.2 Timescales for diffusion in aerosol particles

It has been proposed that some types of atmospheric aerosol particle may be present in the form of a glass or semi-solid (*e.g.* a gel) over a wide range of temperature and relative humidity conditions.^70,78–85^ The phase states of aerosol populations have been investigated using impactors, whereby the fraction of particles which rebound from a surface is used to determine a bouce factor – the higher the bounce factor, the more solid the particles are inferred to be.^86^ The viscosity of proxies for organic aerosol has been reported by a number of authors, including ref. 67, 85 and 87–89, but diffusion measurements are lacking for most materials. Quantitative information about how molecules diffuse in solutions relevant to atmospheric aeorsol is key to predicting how these particles will evaporate and interact with gas phase species *via* multiphase and heterogeneous chemistry.

**Fig. 10:**
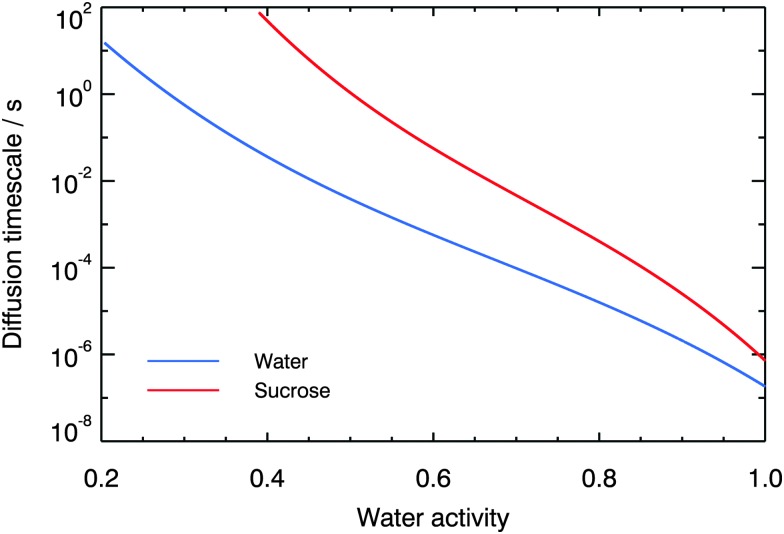
Diffusion timescales for sucrose and water molecules in aqueous sucrose at 296 K, as a function of water activity, predicted using fits to the diffusion coefficient data and eqn (6).

To approximate the diffusion timescales of small and large molecules within aqueous aerosol particles, we calculate the characteristic half-time for diffusion into a spherical particle of radius *r* at constant water activity using:^90^
6
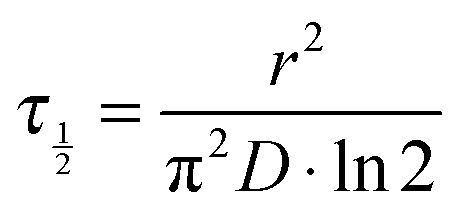
 It can be seen from Fig. 10 that the approximate room temperature diffusion timescales of water and sucrose in aqueous sucrose deviate by nearly four orders of magnitude at 40% RH. Consequently, the diffusion timescales calculated according to eqn (6) also vary by nearly four orders of magnitude: for a 100 nm diameter particle, the half-time for sucrose diffusion is ∼100 s, whereas water diffusion occurs on timescales much faster than 1 s.

If the diffusion of organic molecules in atmospheric aerosol is similar to that of sucrose in aqueous sucrose, then these long timescales could have important implications for particle-phase chemistry and the kinetics of gas-particle partitioning. Slow diffusion of reactants between the bulk of an aerosol particle and its surface could inhibit oxidation. The slow diffusion of large molecules which condense from the vapour phase onto aerosol particles could lead to radial inhomogeneities in the concentrations of different sized molecules. Smaller molecules would be preferentially able to diffuse into the bulk of a particle, whilst larger ones are are unable to diffuse from the surface inwards. Similarly, there may be a kinetic limitation to the evaporation of large organic molecules because they are slow to diffuse from the interior of a particle to its surface. To fully understand this requires the application of a multi-layer kinetics model, which is beyond the scope of this work.

### 3.3 Comparison of diffusion coefficients, viscosity and rebound in aqueous sucrose

**Fig. 11:**
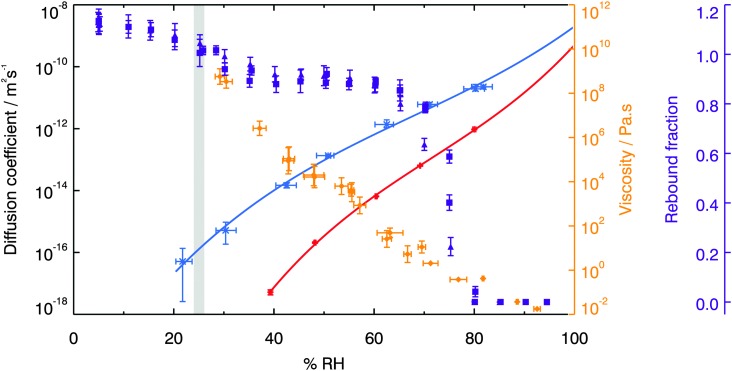
Room temperature diffusion coefficients for water (blue crosses) and sucrose (red plus signs) in aqueous sucrose, plotted against relative humidity and compared with viscosity measurements from Power *et al.* ^67^ (orange diamonds) and rebound fractions measured by Bateman *et al.* ^91^ (purple squares for 190 nm particles, purple triangles for 240 nm particles). The grey shaded region represents the glass transition.

Bateman *et al.* ^91^ use impaction apparatus to measure the rebound fraction of aqueous sucrose droplets at room temperature as a function of RH. In light of the new diffusion measurements presented above, it is now possible to compare this rebound with diffusion coefficients for water and sucrose, and viscosity, as shown in Fig. 11. Two transitions in rebound fraction can be observed, where one is situated at ∼25% RH and the other between 70 and 75% RH. The slight decrease in rebound fraction at ∼25% RH apparently corresponds to the glass transition (highlighted in grey). The sharp decrease at 70–75% RH, where the diffusion coefficient of water is ∼10^–11^ m^2^ s^–1^, the diffusion coefficient of sucrose is ∼10^–13^ m^2^ s^–1^ and the viscosity is ∼5 Pa s, occurs where the solution is highly fluid. To further interpret the detailed rebound fraction behaviour and to build a better quantitative understanding of its implications for diffusion, it will be necessary to obtain data of the detailed rheological response for aqueous sucrose solutions over the full RH range.

## 4 Summary

We report measurements of sucrose diffusion coefficients in aqueous solution between water activities of 0.4 and 0.8 at room temperature. These diffusion coefficients are significantly lower than those of water in the same material under the same conditions, and subsequently the diffusion timescales of sucrose are predicted to be much larger than those of water. In aqueous sucrose, the Stokes–Einstein equation was found to be much more successful in predicting organic diffusion than water diffusion using viscosity data. We find that a fractional Stokes–Einstein equation is able to reproduce the diffusion data for both sucrose and water. The fractional exponent is close to 1 for sucrose, but significantly lower for water, demonstrating the larger decoupling observed for water compared with sucrose. The use of this relationship may pave the way for predicting water diffusion from measurements of viscosity.

The measurements of water and sucrose diffusion in aqueous sucrose presented here are the first of their kind in this binary solution at high solute concentration. They therefore provide a valuable means to study diffusion in a simple but widely used material. Future work should focus on comparing these results with rotational diffusion, relaxation and translational diffusion of other molecules in this material, in order to discern more information about the fundamental nature of diffusion.

Datasets associated with this work (including all Raman spectra, humidity and temperature data and calculated sucrose diffusion coefficients) are provided in Price *et al.* ^92^
